# Ferroptosis-mediated intestinal decolonization of *Klebsiella pneumoniae* using Fe/PPy nanomaterials under near-infrared light

**DOI:** 10.3389/fmicb.2026.1749597

**Published:** 2026-02-20

**Authors:** Xu Zhang, Zelin Yan, Binna Zhang, Siyu Shi, Yuchen Wu, Yanyan Zhang, Danxia Gu, Huqiang Tang, Rong Zhang

**Affiliations:** 1 Department of Clinical Laboratory, The Second Affiliated Hospital Zhejiang, University School of Medicine, Zhejiang, China; 2 The Second School of Clinical Medicine, Anhui Medical University, Hefei, Anhui, China; 3 Centre of Laboratory Medicine, Zhejiang Provincial People's Hospital, People's Hospital of Hangzhou Medical College, Hangzhou, China

**Keywords:** ferroptosis, intestinal decolonization, *Klebsiella pneumoniae*, near-infrared light, reactive oxygen species

## Abstract

**Introduction:**

Klebsiella pneumoniae (KP) is a Gram-negative bacterium with a thick capsule that confers natural drug resistance, making it a common opportunistic pathogen. In recent years, the spread of *hypervirulent Klebsiella pneumoniae* (hvKP) and carbapenem-resistant Klebsiella pneumoniae (CR-KP) strains has created major treatment challenges. Among these, of particular concern is *carbapenem-resistant hypervirulent Klebsiella pneumoniae* (CR-hvKP), which combines both hypervirulence and carbapenem resistance. This strain can persistently colonize the gut, facilitating resistance gene spread and causing bacterial translocation with subsequent infections. CR-hvKP has now become a key pathogen in both hospital and community settings. Traditional antibiotic treatments often lead to the emergence of bacterial resistance, necessitating the development of novel antimicrobial strategies.

**Methods:**

This study introduces an iron-polypyrrole nanocomposite (Fe/PPy) designed to leverage an innovative mechanism of ferroptosis-induced bacterial killing. The nanomaterial possesses intrinsic Fenton reaction activity. Moreover, its photothermal conversion efficiency exceeds 85%. Under 1,064 nm near-infrared (NIR) light, its photothermal effect significantly enhances the Fenton reaction efficiency, thereby effectively catalyzing the conversion of bacterial endogenous H₂O₂ into reactive oxygen species (ROS), thereby inducing bacterial lipid peroxidation and achieving targeted bacterial killing via the ferroptosis pathway.

**Results:**

The excellent tissue penetration of 1,064 nm NIR light enables this material to act precisely on deep-seated infectious lesions, achieving *in vivo* antibacterial efficacy. Experimental results demonstrate that Fe/PPy nanomaterials exhibit high antimicrobial efficacy and safety against KP without readily inducing bacterial resistance.

**Discussion:**

This study provides a novel nanodrug design approach for the clinical treatment of KP infections, offering significant translational potential in the field of anti-infective therapy.

## 1 Introduction

*Carbapenem-Resistantklebsiella pneumoniae* (CR-KP) and *Hypervirulentklebsiella pneumoniae* (hvKP) are significant public health concerns due to their association with high mortality rates (Fang et al., 2022; Russo and Marr, 2019; Wang Q. et al., 2024; Yang et al., 2022). Sequence type ST11 is the predominant clone, linked to infections with a notably high in-hospital mortality rate of 33. 5% (Russo and Marr, 2019). Meanwhile, ST23 hvKp organisms have acquired various antimicrobial resistance (AMR) genes but have not lost key virulence-associated genes, maintaining their strong pathogenicity (Chang et al., 2022). CR-KP strains exhibit resistance to carbapenem antibiotics, limiting effective treatment options and leading to increased patient mortality (Jia et al., 2024; Pu et al., 2023; Pu et al., 2023; Yang et al., 2021) hvKP strains possess enhanced virulence factors, resulting in severe infections and elevated mortality rates. The emergence of these pathogenic strains underscores the urgent need for novel antimicrobial strategies to combat these life-threatening infections (Wang R. et al., 2024). In clinical practice, when KP colonizes the intestine, it can spread to other parts of the body through the bloodstream, causing systemic infections such as pneumonia and sepsis (Choby et al., 2020; Zhang Q. et al., 2023; Osbelt et al., 2021; Xie et al., 2025). Traditional approaches primarily rely on antibiotics to decolonize the intestine; however, the rapid evolution of hvKP and the widespread prevalence ofCR-KP, along with the persistent biofilm formation by these bacteria, significantly reduce the penetration of drugs (Miller and Arias, 2024; Eller et al., 2021; Bengoechea and Sa Pessoa, 2019; Siu et al., 2012). These factors present certain limitations in clinical treatment. Additionally, antibiotic therapy itself may disrupt the intestinal barrier, inadvertently promoting the intestinal translocation of CR-KP (Gu et al., 2018). In an ICU of a tertiary hospital in China, a KP strain with dual resistance to carbapenems and tigecycline, which had been persistently colonizing the intestines of patients, posed a considerable challenge for clinical management (Liu et al., 2022). Meanwhile, alternative therapies, such as probiotics, can be employed to modulate the intestinal microbiota, but their effectiveness in eliminating resistant bacterial colonization remains limited (Fang et al., 2022; Hu et al., 2024; Chen et al., 2021). These challenges underscore the urgent need for innovative therapeutic strategies that bypass conventional resistance mechanisms while maintaining efficacy against CR-KP (Hu et al., 2024).

Nanomedicine has emerged as a revolutionary frontier in antimicrobial therapy, offering a versatile platform for precise targeted treatment and multimodal synergistic interventions (Zhang X. et al., 2023). Drawing from our group’s previous oncology research, we developed an ultrasmall Fe/PPy nanomaterialscapable of selectively inducing tumor cell death throughferroptosis (Li et al., 2021). This nanomaterials exhibits dual functional mechanisms: (i) As a photothermal conversion agent, it achieves a NIR light-to-heat conversion efficiency exceeding 85%, enabling spatiotemporally controlled thermal effects in the range of 42–50 °C, effectively ablating tumor cells;(ii) Owing to its iron-rich nature, this component continuously generates ROS via the Fenton reaction, significantly amplifying oxidative stress levels within the tumor microenvironment (Zhang X. et al., 2023).

Although Fe/PPy demonstrated enhanced antibacterial activity against *Klebsiella pneumoniae* in this study, this effect does not imply absolute species-specific killing. Instead, the observed antibacterial behavior is more appropriately described as a preferential or potentially selective effect. Previous studies have shown that different bacterial species exhibit substantial differences in membrane composition, surface charge distribution, and outer membrane architecture (Makvandi et al., 2023; Kim et al., 2018). These physicochemical differences may influence nanoparticle adsorption efficiency as well as the local accumulation and bioavailability of Fe^3+^ ions released from the Fe/PPy system, thereby contributing to differential antibacterial outcomes among bacterial species.

This study is based on the presence of endogenous hydrogen peroxide (H₂O₂) in bacteria, a key feature of microbial redox metabolism. Endogenous H₂O₂ primarily arises from the incomplete reduction of oxygen during cellular metabolism. As a common byproduct of bacterial energy metabolism, it is widely present across diverse bacterial taxa and is not unique to any single species (Imlay, 2008; Imlay, 2013). Given the role of endogenousH₂O₂ in bacteria, we explored the potential antibacterial mechanism of Fe/PPy nanomaterials to understand how to utilize these reactive substances for catalytic effects. Importantly, we recognize that Fe/PPy nanomaterials may not directly penetrate the bacterial cell wall to exert their antibacterial action (Xue et al., 2023). The mechanism is more likely as follows: Fe/PPy nanoparticles first adsorb onto the bacterial surface through electrostatic interactions, releasing Fe^3+^ upon irradiation with NIR light; subsequently, the Fe^3+^ enters the bacterial interior, where it reacts with endogenous H₂O₂in a Fenton reaction, catalyzing the generation of hydroxyl radicals (•OH) and thus inducing bacterial ferroptosis (Guerrieri et al., 2021; Fromain, 2023). Recent investigations reveal that Fe/PPy’s ferroptosis-inducing capability extends beyond eukaryotic cells (Chen et al., 2021; Dixon et al., 2012; Gan et al., 2023). When repurposed for bacterial eradication, the nanomaterial triggers iron dyshomeostasis and lipid peroxidation cascades in KP, effectively inducing bacterial ferroptosis. This bactericidal mechanism operates independently of traditional antibiotic targets, thereby circumventing existing resistance pathways (Blair et al., 2015). Furthermore, the concomitant photothermal effect disrupts biofilm integrity through localized hyperthermia, sensitizing persister cells to ferroptotic stress (Fulaz et al., 2019). Such multimodal synergy—combining physical biofilm disruption, metabolic perturbation via glucose oxidase (GOX)-mediated starvation, and ferroptosis induction—represents a paradigm shift in antimicrobial strategy (Huo et al., 2025; Bankar et al., 2009; Jiang et al., 2021). Notably, recent studies have demonstrated that nanomaterials can effectively eradicate bacteria through the ferroptosis pathway (Liu et al., 2023; Cui et al., 2023; Cui et al., 2023). Inspired by these findings, we engineered an established cancer therapeutic nanoplatform for antimicrobial applications, leveraging its intrinsic ferroptosis-inducing capability while incorporating photothermal synergistic effects (Cui et al., 2023). This work not only unveils a previously unrecognized bacterial ferroptosis pathway but also establishes a blueprint for cross-disciplinary therapeutic innovation. Fe/PPy can concurrently address biofilms, virulence factors of hypervirulent *Klebsiella pneumoniae*, and multiple resistance mechanisms, making it a promising translational candidate in precision nanomedicine and offering important implications for the treatment of recalcitrant bacterial infections.

## 2 Methods

### 2.1 Materials

*Synthesis of Fe₃O₄ Nanoparticles (NPs):* First, FeCl₃ (324 mg, 2.0 mmol) was dissolved in diethylene glycol (DEG, 20 mL) under vigorous stirring until a homogeneous solution was obtained. Subsequently, sodium citrate (206 mg, 0. 8 mmol) and sodium acetate (NaOAc, 492 mg, 6.0 mmol) were sequentially added, with continuous stirring until complete dissolution. The resulting mixture was then transferred into a polytetrafluoroethylene-lined autoclave and subjected to hydrothermal treatment at 210 °C for 10 h. After cooling to room temperature, the obtained product was thoroughly washed with deionized water and ethanol several times to remove residual reactants. Finally, small-sized magnetic Fe₃O₄ NPs were successfully obtained.

*Synthesis of Fe/PPy NPs:* Fe₃O₄ NPs (5.6 mg), prepared as described above, were dispersed in a polyvinylpyrrolidone (PVP, 5 mL) solution and ultrasonicated to achieve uniform dispersion. Subsequently, N, N-dimethylformamide (DMF, 200 μL), pyridine (py, 5 mL), and carboxylated pyridine (py-COOH, 10 mg) were added, followed by ultrasonic treatment to facilitate dissolution and homogeneous mixing. Hydrochloric acid solution (HCl, 200 μL, 2 mol/L) was then introduced into the reaction system, which was maintained at 37 °C for 24 h. Upon completion of the reaction, the product was purified through alternating ethanol and water washes, collected by centrifugation, and finally redispersed in ultrapure water to yield Fe/PPy NPs.

*Monitoring of extracellular photothermal effect:* Fe/PPy aqueous solutions with different concentrations (25, 50, 75, and 100 ppm) were first prepared. The samples were then irradiated with a NIR light at a wavelength of 1,064 nm (2 W/cm^2^), while a high-precision thermocouple thermometer was employed to record real-time temperature variations of the solution. This approach systematically evaluated the photothermal conversion performance of the material.

*Detection of ·OH:* To quantitatively detect the generation of ·OH during the reaction, MBwas used as a specific indicator. The detection principle is based on the ability of ·OH to selectively oxidize and degrade MB molecules, resulting in a gradual decrease in the intensity of the characteristic absorption peak as the reaction progresses. First, Fe/PPy material, MB solution (200 μM), and hydrogen peroxide (H₂O₂, 10 mM) were mixed in the predetermined ratio and irradiated with NIR for 1,064 nm (2 W/cm^2^, 10 min). After the reaction, the full spectrum was scanned using a UV–Vis spectrophotometer, and the absorbance at the characteristic MB absorption wavelength (660 nm) was recorded, enabling the detection of hydroxyl radicals.

*Evaluation of Fe/PPy:* To systematically evaluate the antimicrobial performance of Fe/PPy, this study employed the plate colony counting method and Syto9/PI live/dead staining assay to test the 22-ZR and HvKP4 bacterial strains. The frozen bacterial strains were first revived and streaked onto LB agar plates, followed by incubation at 37 °C overnight. Single colonies were then picked and inoculated into LB liquid medium, which was cultured at 37 °C with shaking at 200 rpm until the logarithmic growth phase was reached. The bacterial suspension was adjusted to a concentration of 1 × 10^7^ CFU/mL using PBS buffer. For the antimicrobial experiment, the bacterial suspension was co-cultured with Fe/PPy material and subjected to NIR treatment for 8 min (1,064 nm, 2 W/cm^2^). The treated bacterial suspension was diluted 10 (Wang Q. et al., 2024)-fold and then plated onto LB agar plates, and incubated at 37 °C for 24 h. CFU were counted to quantitatively evaluate the antimicrobial effect. Additionally, the Syto9/PI dual staining kit was used to further analyze bacterial viability. After treatment, the bacterial suspension was centrifuged at 4000 rpm for 10 min, and the pellet was resuspended in Syto9/PI mixed staining solution and incubated in the dark for 10 min. Finally, a confocal microscope was used to observe and record the distribution of live bacteria (green fluorescence) and dead bacteria (red fluorescence), providing a visual representation of the antimicrobial performance of the material.

*In vitro antibiofilm properties of Fe/PPy:* To comprehensively evaluate the inhibitory effect of Fe/PPy on bacterial biofilms, this study combined crystal violet staining quantification with SEMmorphology observation. Initially, the bacterial strains were cultured statically for 48 h to allow for the formation of mature biofilms. After co-culturing with Fe/PPy material, the biofilms were subjected to NIR treatment for 8 min (1,064 nm, 2 W/cm^2^). The supernatant was then removed, and the biofilms were stained with 0. 1% crystal violet solution for 30 min to assess the degree of biofilm disruption. In addition, to visually observe the structural changes in the biofilm, the treated samples were centrifuged at 4000 rpm for 10 min. The pellets were fixed overnight at 4 °C with 2. 5% glutaraldehyde, followed by dehydration through a gradient ethanol series (30, 50, 70, 80, 90, 95, and 100% ethanol, each for 15 min). After critical point drying and gold sputtering, the samples were examined using a field-emission scanning electron microscope at various magnifications. This allowed for the observation of the biofilm’s three-dimensional structure and surface morphology, providing a systematic analysis of the disruption caused by Fe/PPy on the biofilm.

*Intracellular ROS detection:* DCFH-DA fluorescence probe was used to detect intracellular ROS levels in bacteria. This probe specifically reacts with ·OH, producing green fluorescence. Initially, the bacterial suspension was co-cultured with Fe/PPy material for 4 h, followed by NIR irradiation for 8 min. After treatment, the bacterial suspension was centrifuged at 4000 rpm for 10 min to collect the bacterial cells. The cells were then resuspended and incubated in DCFH-DA fluorescence staining solution in the dark for 30 min. After staining, the bacteria were washed with PBS buffer to remove any unbound dye. Finally, a confocal microscope was used to observe and record the intensity of the green fluorescence within the bacteria, which serves as an indicator of intracellular ROS generation.

*Measurement of lipid peroxidation:* Lipid peroxidation in bacterial cells was evaluated by quantifying MDA levels using a commercial MDA assay kit. Briefly, hvKP4 and 22ZR-42 were subjected to different treatments (Control, NIR, Fe/PPy, Fe/PPy + NIR, and Fe/PPy + NIR + DFO). After treatment, bacterial samples were collected and lysed, and equal volumes of the lysates were mixed with MDA working solution. The mixtures were heated at 100 °C for 15 min to allow the thiobarbituric acid (TBA) reaction to proceed. After cooling to room temperature, the samples were centrifuged, and the supernatants were transferred to a 96-well plate. Absorbance was measured at 532 nm using a microplate reader. MDA levels were calculated based on a standard curve and normalized to the total protein content of each sample.

*Hemolysis assay: H*emolysis assays were performed using EDTA-anticoagulated whole blood from C57BL/6 female mice. Female mice were anesthetized by intraperitoneal administration of tribromoethanol (250 mg/kg), followed by blood collection via the enucleation method. The blood samples were centrifuged at 3000 rpm for 5 min, and the supernatant (containing serum, platelets, and the white blood cell layer) was discarded. The remaining red blood cells (RBCs) were washed three times with PBS buffer and finally resuspended to prepare a 4% RBC suspension. The experiment included a negative control (PBS) and a positive control (Water). These controls, along with different concentrations of Fe/PPy nanomaterials, were co-incubated with the RBC suspension for 24 h at 37 °C under 5% CO₂ conditions. After incubation, the samples were centrifuged at 3000 rpm for 5 min, and the supernatant was collected. The absorbance of the supernatant was measured at 576 nm using a microplate reader. The hemolysis rate was calculated using the following formula:


$$\begin{array}{l} \text{Hemolysis rate}\;(\%)=[({\mathrm{OD}}_{576}\text{sample}-{\mathrm{OD}}_{576}\text{negative control})\\ /({\mathrm{OD}}_{576}\text{positive control}-{\mathrm{OD}}_{576}\text{negative control})]\times 100\% \end{array}$$

*Complete blood count and blood biochemical analysis: B*lood samples were collected from C57BL/6 female mice on days 1 and 7 post-administration using EDTA- and heparin-anticoagulated tubes. Female mice were anesthetized by intraperitoneal administration of tribromoethanol (250 mg/kg), and blood collection via the enucleation method. EDTA-anticoagulated blood was analyzed using an automated hematology analyzer for complete blood count (CBC). Heparin-anticoagulated blood was centrifuged at 3000 rpm for 5 min to separate plasma, which was then used for biochemical analysis.

*In vivo De-Colonization effect evaluation:* C57BL/6 female mice were used to establish an intestinal colonization model. Bacterial suspensions in the logarithmic growth phase (cultured in LB medium) were adjusted to 1 × 10^7^ CFU/mL, and 100 μL was administered via oral gavage. After successful colonization, Fe/PPy nanomaterials were orally administered for intervention, followed by NIR irradiation of the abdominal region after 4 h (1,064 nm, 2 W/cm^2^, 8 min). After 24 h of intervention, fecal samples were collected for 16S rRNA sequencing analysis to assess gut microbiota changes. The female mice were subsequently euthanized by cervical dislocation, and the heart, liver, spleen, lungs, kidneys, and intestinal tissues were harvested for hematoxylin–eosin (H&E) staining and pathological evaluation.

## 3 Results and discussion

### 3.1 Antimicrobial susceptibility profiles of HVKP4 and 22ZR-42

The antimicrobial susceptibility testing results (Table 1 and Supplementary Figure S1) revealed that strain HVKP4 exhibited extensive drug resistance to the tested antibiotics, demonstrating susceptibility only to tigecycline (MIC = 0. 5 mg/L) and polymyxin B (MIC ≤ 0.5 mg/L). It showed resistance to all tested *β*-lactam antibiotics, including meropenem, ertapenem, and ceftazidime. In contrast, the resistance profile of strain 22ZR-42 showed significantly higher susceptibility to carbapenemsand cephalosporinscompared to HVKP4. However, 22ZR-42 remained completely resistant to cefotaxime and aztreonam. The findings demonstrate that both clinical isolates of KP exhibited severe multidrug-resistant phenotypes, with markedly limited therapeutic options among existing antimicrobial agents. This phenomenon underscores the urgent need to develop novel antibacterial drugs to combat infections caused by such resistant strains.

**Table 1:** Antimicrobial susceptibility profiles of HVKP4 and 22ZR-42.

Antibiotic	HVKP4 (MIC, mg/L)	22ZR - 42 (MIC, mg/L)
IMP	**16**	**16**
MEM	**128**	**32**
ETP	**>128**	**64**
CMZ	**128**	8
CAZ	**>128**	**32**
CTX	**>128**	**>128**
TZP	**>256/4**	**>256/4**
SCF	**>256/128**	**128/64**
CAV	2/4	≤0. 5/4
FEP	**>64**	**>64**
PB	≤0. 5	1
TGC	**0. 5**	≤0. 25
CIP	**32**	≤1
AK	**>128**	≤4
ATM	**>128**	**>128**

IMP, imipenem; MEM, meropenem; ETP, ertapenem; CMZ, cefmetazole; CAZ, ceftazidime; CTX, cefotaxime; TZP,piperacillin/tazobactam; SCF, cefoperazone/sulbactam; CAV, ceftazidime/avibactam; FEP, cefepime; PB, polymyxin B; TGC, tigecycline; CIP, ciprofloxacin; AK, amikacin; ATM, aztreonam. *The MIC for resistance was used bold font (mg/L).

### 3.2 Synthesis and characterization of Fe/PPy

**Figure 1:**
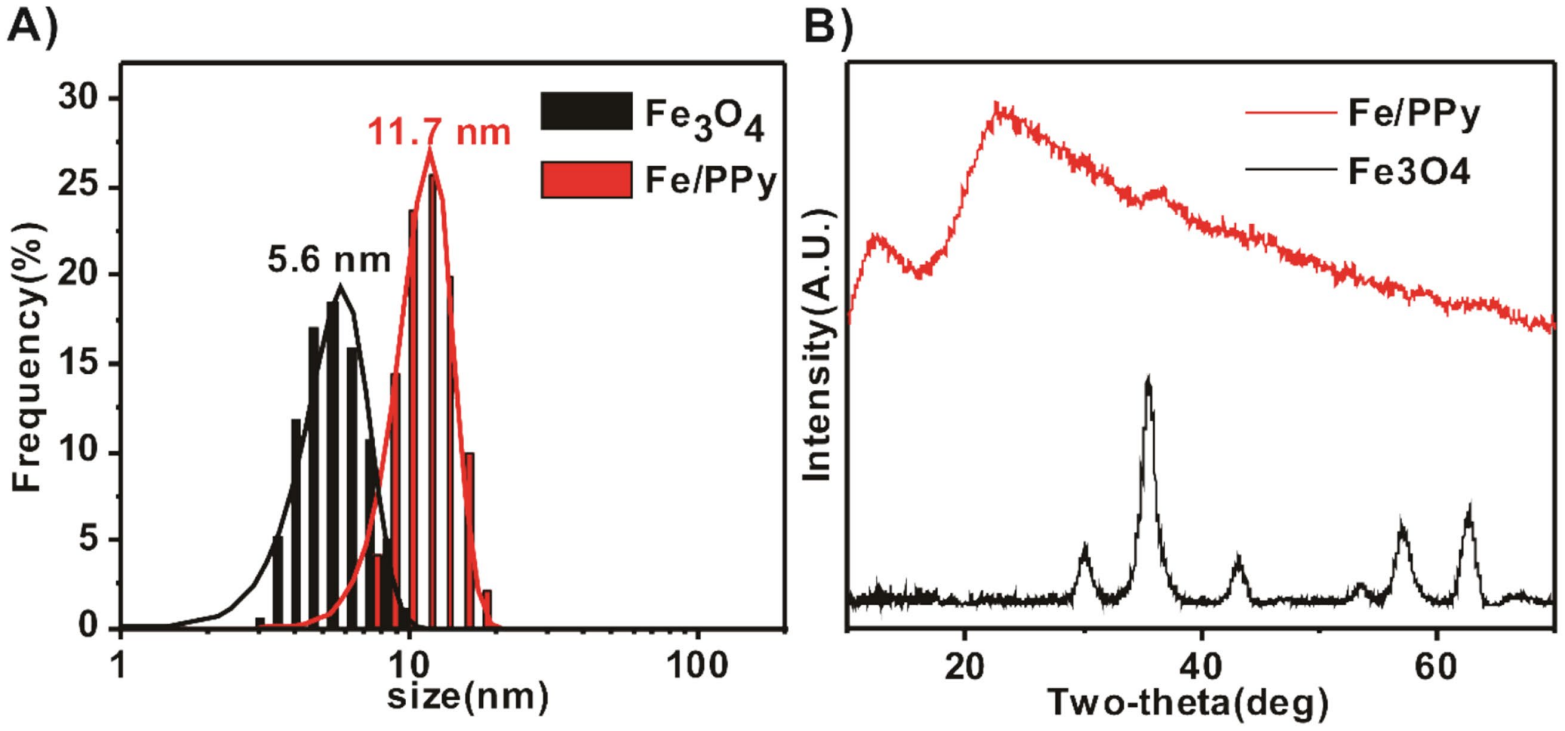
Characterization of Fe_3_O_4_ nanoparticles and Fe/PPy nanocomposites. **(A)** Particle size distribution statistics of Fe_3_O_4_ and Fe/PPy. **(B)** X-ray diffraction (XRD) patterns of Fe_3_O_4_ (JCPDS no. 19-0629) and Fe/PPy.

We successfully synthesized Fe/PPy using a one-pot method (Zhang X. et al., 2023). The dynamic light scattering (DLS) measurements show the hydrodynamic diameters of Fe_3_O_4_ and Fe/PPy to be 5 nm and 7 nm (Figure 1A). This discrepancy in particle size characterization may arise from the solvation effect between the hydrophilic functional groups on the particle surface and water molecules, leading to the formation of a hydration layer on the particle surface during DLS detection (Kurzbach et al., 2013). As a result, the hydrodynamic diameter measured by DLS is slightly larger than the crystallographic size observed by TEM. The XRD characterization results show that the diffraction peaks of the synthesized Fe_3_O_4_ nanoparticles are consistent with the standard card (JCPDS no. 19–0629). In the Fe/PPy composite material, however, the characteristic peaks of Fe_3_O_4_ completely disappear, and only two broad diffraction bands appear in the 10–30° range, which is consistent with the amorphous structure of polypyrrole. This indicates that the hydrochloric acid etching process has completely removed the Fe_3_O_4_ core (Figure 1B).

### 3.3 Photothermal properties and reactive oxygen species generation analysis

**Figure 2:**
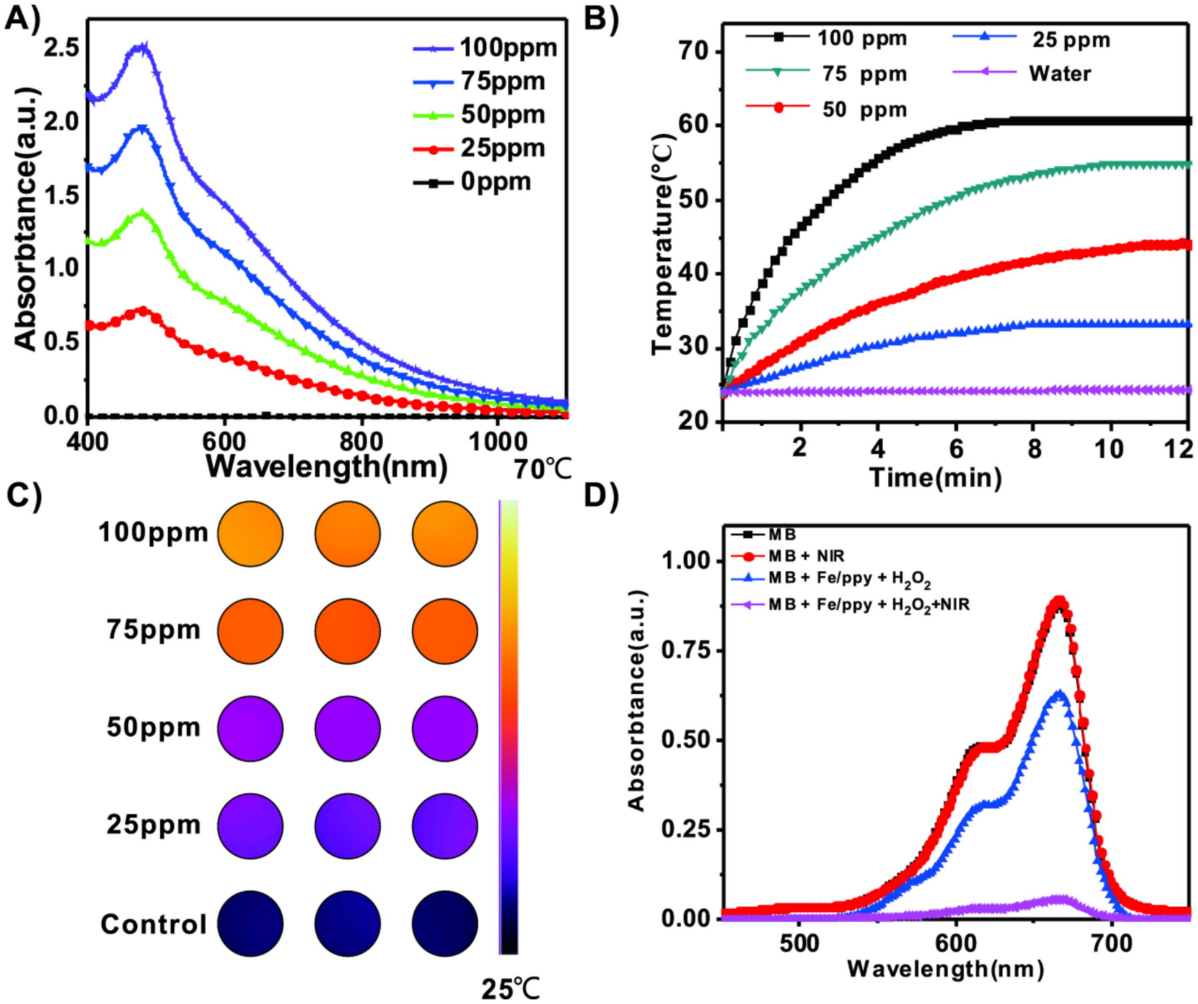
Photothermal properties and reactive oxygen species generation analysis. **(A)** UV–Vis absorption spectrum of Fe/PPy. **(B)** Temperature rise curves of Fe/PPy at different concentrations under 1,064 nm laser irradiation. **(C)** Photothermal infrared thermal images of samples at corresponding concentrations. **(D)** Changes in absorbance of MBsolution under different treatment conditions.

The UV–Vis–NIR absorption spectra revealed that Fe/PPy exhibited a broad and strong absorption band centered around 1,064 nm, providing a theoretical basis for its application in NIR light therapy (Figure 2A). To systematically evaluate the photothermal conversion performance of the material, we measured the temperature changes of Fe/PPy dispersions at different concentrations (25–100 ppm) under 1,064 nm NIR light irradiation (1.0 W/cm^2^, 15 min). The results showed a clear concentration-dependent heating effect, with the highest temperature reaching 58. 3 ± 1. 2 °C (100 μg/mL), significantly higher than the PBS control group (Figure 2B). Infrared thermal imaging further confirmed the excellent photothermal conversion performance of Fe/PPy (Figure 2C and Supplementary Figure S2). To investigate its antimicrobial mechanism, we assessed the generation of hydroxyl radicals (OH) using a methylene blue (MB) decolorization assay. In a Fenton reaction system, Fe/PPy effectively catalyzed the decomposition of H₂O₂ under NIR light, generating ·OH and leading to a significant reduction in the characteristic MB absorption peak (664 nm). In contrast, the MB degradation rate in the NIR + H₂O₂ and Fe/PPy + H₂O₂ groups was considerably lower than that in the Fe/PPy + NIR + H₂O₂ group (Figure 2D). These findings confirm that Fe/PPy + NIR can efficiently generate reactive oxygen species through photothermal-enhanced Fenton reactions, demonstrating its potential to induce bacterial ferroptosis.

### 3.4 Antimicrobial activity of Fe/PPy

**Figure 3:**
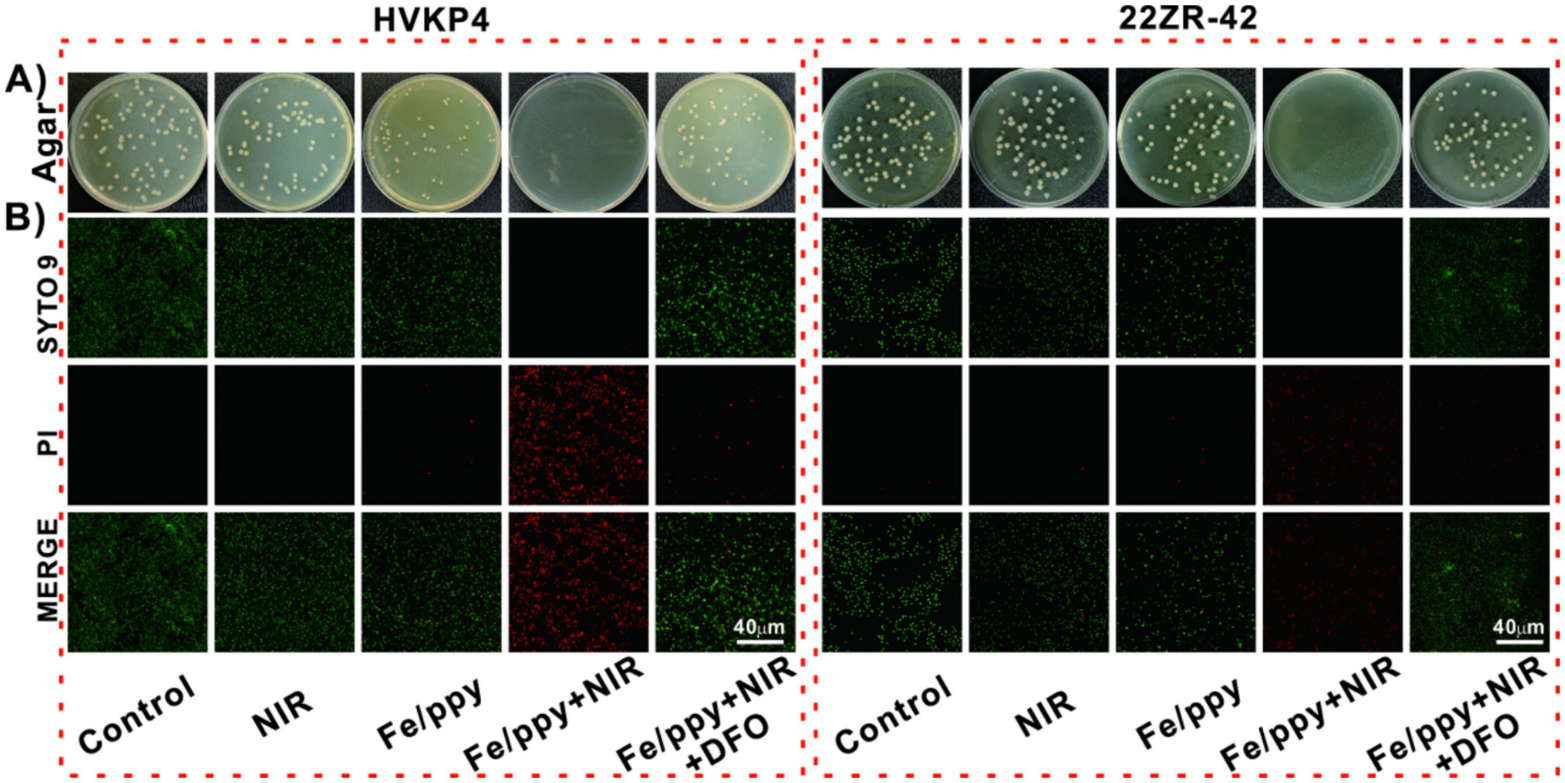
Analysis of the antimicrobial performance of Fe/PPy nanocomposites. **(A)** Plate coating experiment results for HvKP4 and 22ZR-42 under different treatment groups (from left to right: control group, NIR group, Fe/PPy group, Fe/PPy + NIR group, Fe/PPy + NIR + DFO group). **(B)** Confocal microscopy images of bacterial live/dead staining (SYTO 9/PI dual staining: green indicates live bacteria, red indicates dead bacteria; scale bar: 40 μm).

The combined treatment of Fe/PPy nanomaterials and NIR light significantly inhibited the growth of KP (HvKP4 and 22-ZR) in colony counting assays (with 100% killing efficiency against bothHvKP4 and 22-ZR). In contrast, no significant difference was observed between the NIR group (19.2 and 12% killing efficiency against HvKP4 and 22-ZR, respectively), the Fe/PPy group (22.1% and 17.9%, respectively), and the control group, confirming the importance of the synergistic treatment. Notably, this potent combined effect was significantly reduced (to 37.8% and 29.9%, respectively) upon addition of the ferroptosis inhibitor DFO (Figure 3A and Supplementary Table S1). The bacterial growth counting results further confirmed the antibacterial effect of the Fe/PPy nanomaterials (Supplementary Figure S3). To visualize the antimicrobial effects of different treatments, a dual staining method using SYTO 9 Green Fluorescent Nucleic Acid Stain (SYTO 9, green fluorescence for live bacteria) and propidium iodide (PI, red fluorescence for membrane-damaged dead bacteria) was applied (Figure 3B). The treated KP strains were then analyzed *in situ* using confocal laser scanning microscopy (CLSM). Three-dimensional CLSM imaging revealed that in the Control, NIR, and Fe/PPy groups, the bacterial colonies predominantly emitted green fluorescence, indicating that single interventions showed no significant antimicrobial activity. In contrast, the Fe/PPy + NIR group exhibited extensive red fluorescence, confirming that the synergistic treatment effectively inhibited bacterial growth. After the addition of the ferroptosis inhibitor DFO, the red fluorescence intensity in the combined treatment group decreased, and the green/red fluorescence area ratio increased, suggesting that the ferroptosis pathway was specifically blocked. Furthermore, quantitative analysis of fluorescence intensity objectively demonstrated the antibacterial efficacy of Fe/PPy nanomaterials (Supplementary Figure S4). These dynamic fluorescence imaging results were in high agreement with the quantitative colony count data, collectively demonstrate the superior antibacterial efficacy of the synergistic treatment.

### 3.5 Ferroptosis antimicrobial mechanism analysis

**Figure 4:**
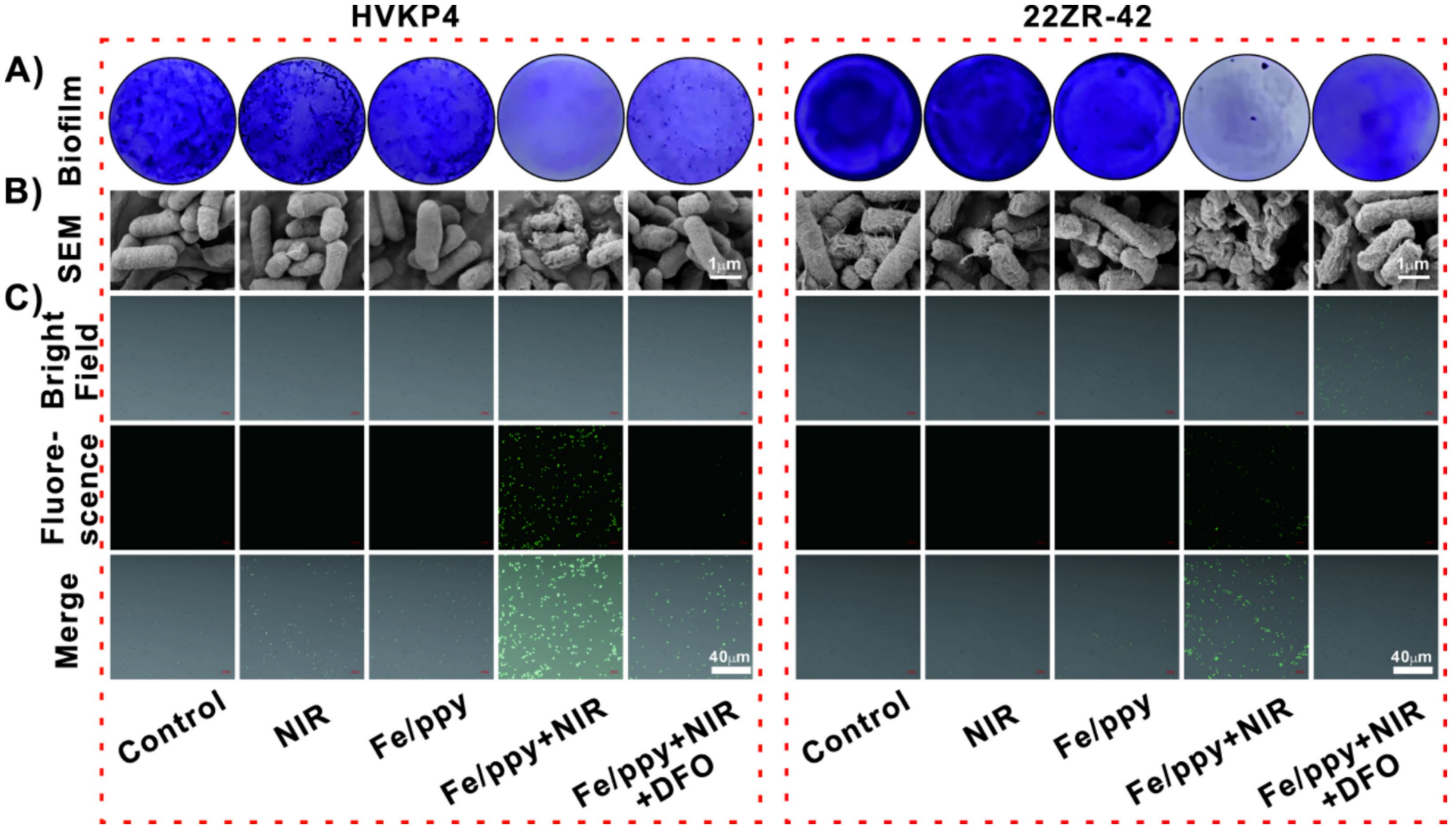
Antimicrobial mechanism analysis of HvKP4 and 22ZR-42 treated with Fe/PPy. **(A)** Quantitative analysis of biofilm formation using crystal violet staining in different treatment groups (from left to right: control group, NIR group, Fe/PPy group, Fe/PPy + NIR group, Fe/PPy + NIR + DFO group). **(B)** SEM observation of the three-dimensional biofilm structure under different treatments (scale bar: 1 μm). **(C)** Intracellular ROS levels detected by DCFH-DA staining (green fluorescence intensity positively correlates with ROS content; scale bar: 40 μm).

Based on the excellent antimicrobial performance exhibited by Fe/PPy nanomaterials, we conducted an in-depth investigation into their antimicrobial mechanism. Crystal violet staining experiments were performed to observe biofilm formation. The results showedthat whilethe Control group presented complete biofilm structures, the biofilms in the NIR and Fe/PPy groups showed signs of slight disruption, though a basic structure remained intact. However, the Fe/PPy + NIR group exhibited significant biofilm disruption, which could be effectively inhibited by the ferroptosis inhibitor DFO (Figure 4A). To quantitatively validate these findings, we subsequently dissolved the stained biofilms and measured the OD_590_ values, obtaining more definitive quantitative evidence (Supplementary Figure S5). SEM further confirmed that the biofilm structure was markedly disrupted in the Fe/PPy + NIR group (Figure 4B). To explore the generation of•OH during the antimicrobial process, we used the DCFH-DA probe to detect ROSlevels inside the bacteria. Fluorescence detection results showed that there was no significant green fluorescence in the control group; both the NIR and Fe/PPy groups exhibited only faint fluorescence signals. In contrast, the Fe/PPy + NIR group exhibited strong green fluorescence signals, which were significantly weakened in the Fe/PPy + NIR + DFO group (Figure 4C). The fluorescence intensity analysis further validated this phenomenon (Supplementary Figure S6). To further determine whether the elevated oxidative stress led to lipid peroxidation, malondialdehyde (MDA) levels were quantified as an indicator of membrane lipid damage. Fe/PPy + NIR induced a significant increase in MDA content in both hvKP4 and 22ZR-42 compared with the Control, NIR, and Fe/PPy groups (Supplementary Figure S7). Notably, the addition of the ferroptosis inhibitor deferoxamine (DFO) markedly attenuated MDA accumulation, indicating that the lipid peroxidation process was iron-dependent. These experimental results demonstrate that Fe/PPy combined with NIR irradiation exerts significant antimicrobial effects by inducing bacterial ferroptosis, as evidenced by elevated reactive oxygen species generation and lipid peroxidation.

### 3.6 Biocompatibility of Fe/PPy

**Figure 5:**
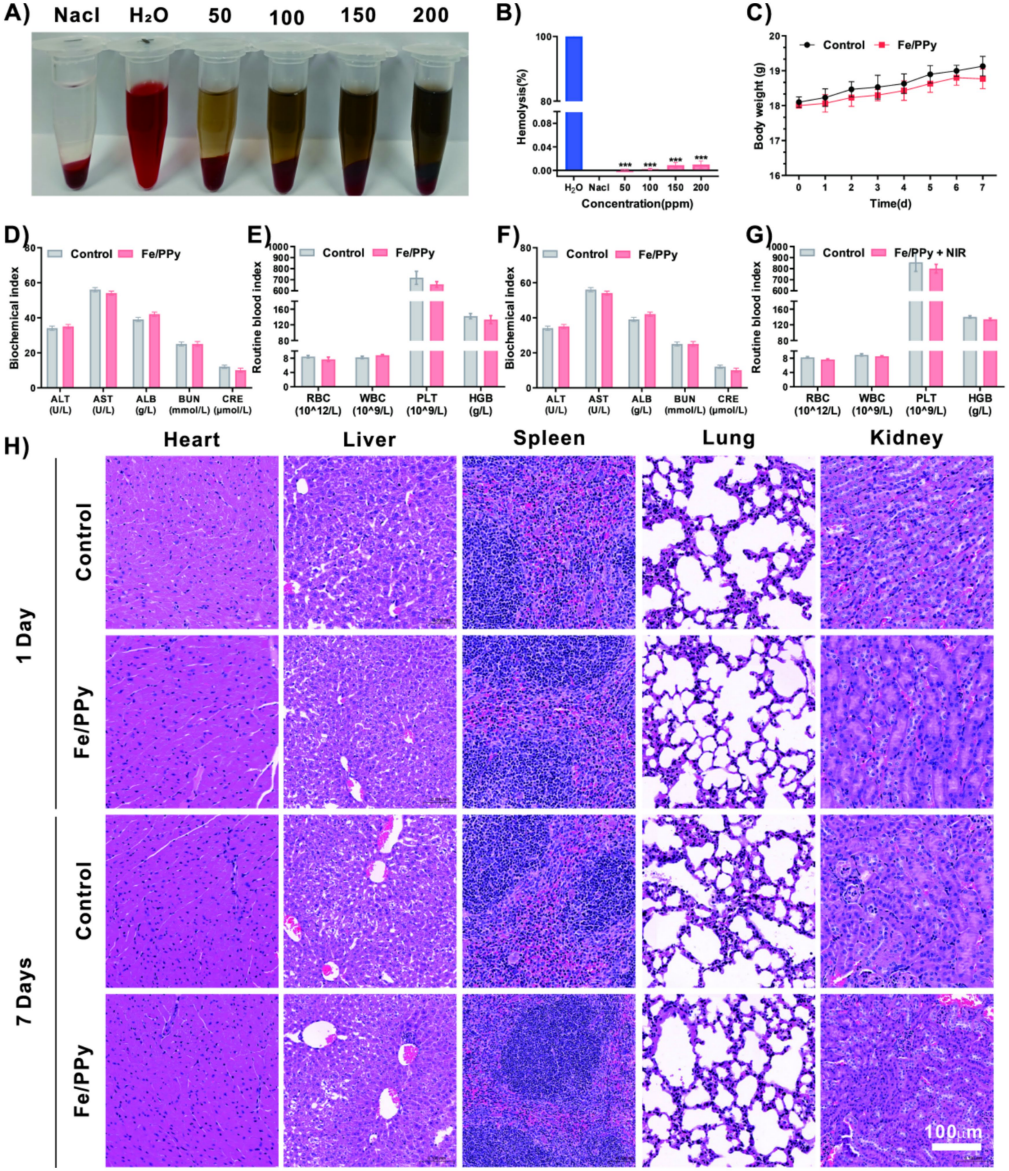
Biological safety evaluation of Fe/PPy nanomaterials. **(A,B)** Hemolysis assay results showing blood sample morphology and quantitative hemolysis rates after treatment with various concentrations of Fe/PPy. **(C)** Body weight change curves of two female mouse groups. **(D,E)** Liver and kidney function indices and blood routine test results 1 day after administration. **(F,G)** Liver and kidney function indices and blood routine test results 7 days after administration. **(H)** Histopathological analysis of major organs via H&E staining at both one-day and seven-day time points (scale bar: 100 μm) (***p < 0.001, **p < 0.01, *p < 0.05).

The biocompatibility of nanomaterials is crucial for assessing their safety in biomedical applications. In this study, the safety of Fe/PPy nanomaterials was comprehensively evaluated through hemolysis and *in vivo* toxicity experiments (Figure 5A). The hemolysis assay showed that the hemolysis rate was below the international standard (<5%), indicating that the material does not cause erythrocyte membrane damage (Figure 5B). *In vivo* experiments were conducted, where animals were orally gavaged with the material, and body weight changes were monitored for 7 days (Figure 5C). The results showed no significant differences between the experimental and control groups (*p* > 0. 05), suggesting that the material did not induce systemic toxicity. To systematically assess the potential impact of the material on the body, blood routine and biochemical analyses were conducted at the appropriate time points post-administration. Blood routine analysis showed that red blood cell (RBC) count, white blood cell (WBC) differential count, and platelet (PLT) parameters were all within normal physiological ranges. Additionally, key liver and kidney function indicators, including Alanine Aminotransferase (ALT), Aspartate Aminotransferase (AST), Albumin (ALB), Blood Urea Nitrogen (BUN), and Creatinine (CRE), were measured. Showed no significant differences between the experimental and control groups (*p* > 0. 05), indicating that the material did not cause any abnormalities in liver and kidney metabolic functions (Figures 5D–G) At the study endpoint, animals were sacrificed, and anatomical observations along with tissue pathological analyses were performed on the major organs (heart, liver, spleen, lungs, kidneys, small intestine). Histological analysis via H&E staining revealed that the organ structures in the experimental groups were intact, with no inflammatory cell infiltration, congestion, edema, or necrosis observed. Notably, liver and kidney tissues, which are closely related to nanoparticle metabolism, maintained normal morphological characteristics, including intact hepatic lobule structures and glomerular filtration barriers. The results from this series of experiments confirm that Fe/PPy demonstrates excellent biocompatibility and safety under the experimental conditions (Figure 5F).

### 3.7 *In vivo* de-colonization of Fe/PPy

**Figure 6:**
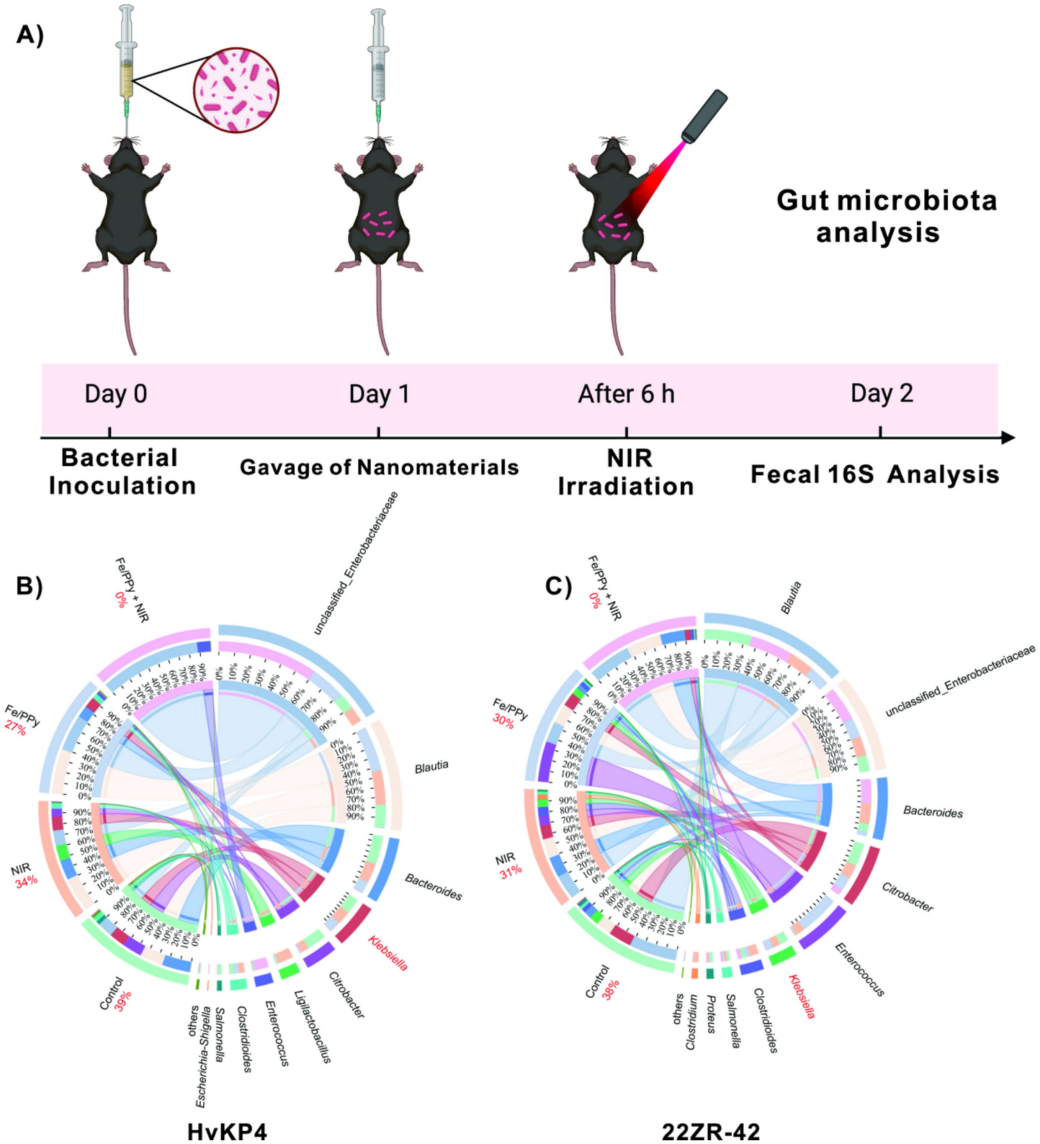
*In vivo* KP decolonization analysis. **(A)** Timeline depicting the establishment of a murine infection model and subsequent treatment with nanomaterials. **(B)** Circos analysis of gut microbiota under various treatment conditions in the 22ZR-42 infection group; **(C)** Circos analysis of gut microbiota under various treatment conditions in the HvKP4 infection group.

Based on the photothermal antimicrobial properties of Fe/PPy and the previously verified biological safety (Figures 4, 5), this study established an intestinal colonization model of KP in C57BL/6 female mice to evaluate the decolonization effect. The model was first established via oral gavage (1 × 10^8^ Colony-forming units, CFU) to induce infections with 22-ZR and HvKP4 strains, followed by treatment (Figure 6A). 24 h post-inoculation, female mice in the Fe/PPy and Fe/PPy + NIR groups were gavaged with an Fe/PPy suspension (20 mg/kg), while the Control and NIR groups were also included (*n* = 5 per group). 4 h later, female mice were anesthetized by intraperitoneal administration of tribromoethanol (250 mg/kg), and were immediately subjected to 1,064 nm NIR irradiation (1.0 W/cm^2^, 8 min) to activate the photothermal effect. This wavelength, compared to 808 nm, provides superior tissue penetration, ensuring effective heating in the intestinal tract. Fecal samples were collected 24 h after treatment, and 16S rRNA high-throughput sequencing was used to analyze the gut microbiota composition. CIRCOS plot analysis revealed that the Fe/PPy + NIR group showed a complete eradication of KP (22-ZR: 0%; HvKP4: 0%), compared to the Control group (22-ZR: 38%; HvKP4: 39%). The NIR group (22-ZR: 31%; HvKP4: 34%) and the Fe/PPy group (22-ZR: 30%; HvKP4: 27%) maintained significant colonization, confirming the necessity of the photothermal synergistic effect (Figure 6B). Additionally, Klebsiella colonization in the intestines of 22-ZR-infected female mice in the Control group was slightly lower than that of HvKP4 (14% vs. 8%), with the HVKP4 strain exhibiting a stronger intestinal residence ability (Supplementary Figure S8). At the experimental endpoint, histological examination of H&E-stained tissues from all treatment groups demonstrated well-preserved tissue morphology in the dissected mice (Supplementary Figure S9).

## 4 Conclusion

We successfully synthesized ultrasmall Fe/PPy nanopolymers with NIR-responsive properties using a hard-template method. Under 1,064 nm NIR irradiation, this material efficiently eliminates *Klebsiella pneumoniae* via a synergistic photothermal–ferroptosis mechanism, achieving significant *in vivo* intestinal decolonization. Mechanistic investigations indicate that the NIR-induced photothermal effect not only contributes to bacterial ablation but, more importantly, promotes iron-dependent lipid peroxidation, thereby triggering bacterial ferroptosis. This dual-action mechanism underlies the high antibacterial efficacy of the Fe/PPy nanoplatform. Benefiting from the superior tissue penetration depth of 1,064 nm NIR light, this strategy shows distinct advantages for the treatment of deep-seated infections caused by multidrug-resistant *K. pneumoniae*. Notably, although Fe/PPy demonstrated enhanced antibacterial efficacy against HvKP4 and 22ZR-42 in the murine intestinal infection model, the molecular basis underlying this preferential susceptibility remains to be fully elucidated. Previous studies have suggested that the unique capsular polysaccharide structures and surface physicochemical properties of *K. pneumoniae* may facilitate nanoparticle adsorption and iron-dependent killing. However, systematic validation of these hypotheses under physiologically relevant polymicrobial conditions is still required. Future studies employing complex gut microbiota models and human-derived samples will be essential to comprehensively clarify the selectivity and translational potential of this nanotherapeutic strategy.

## Data Availability

The original contributions presented in the study are included in the article/Supplementary material, further inquiries can be directed to the corresponding author.
