# Supplementary material for: Ferroptosis-mediated intestinal decolonization of Klebsiella pneumoniae using Fe/PPy nanomaterials under near-infrared light
Source: Front Microbiol. 2026 Feb 20;17:1749597. doi: 10.3389/fmicb.2026.1749597 (PMC12963007; doi:10.3389/fmicb.2026.1749597)
Supplement: Supplementary file 1 [file Data_Sheet_1.PDF]

Supporting Information

Table S1: Antimicrobial efficacy of different treatment groups against HVKP4 and 22ZR-42

Treatment Group	Strain	Mean Colony Count(CFU/ml)	Antibacterial Rate(%)
**Control**	HVKP4	6.8×10^{6}	0
	22ZR-42	6.7×10^{6}	0
**NIR**	HVKP4	5.5×10^{6}	19.2
	22ZR-42	5.9×10^{6}	12
**Fe/PPy**	HVKP4	5.3×10^{6}	22.1
	22ZR-42	5.5×10^{6}	17.9
**Fe/PPy + NIR**	HVKP4	0	100
	22ZR-42	0	100
**Fe/PPy + NIR + DFO**	HVKP4	4.3×10^{6}	37.8
	22ZR-42	4.7×10^{6}	29.9

A)

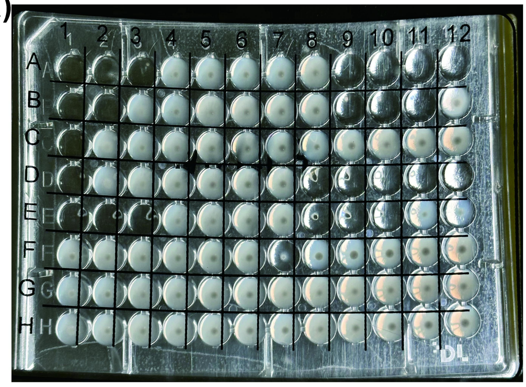

HVKP4

B)

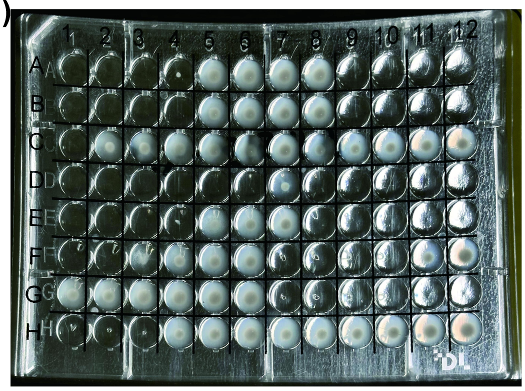

22ZR-42

C)

CRE Antimicrobial Susceptibility Testing Panel (2025)

	1	2	3	4	5	6	7	8	9	10	11	12
A	IPM 128	IPM 64	IPM 32	IPM 16	IPM 8	IPM 4	IPM 2	IPM 1	CAV 64/4	CAV 32/4	CAV 16/4	CAV 8/4
B	MEM 128	MEM 64	MEM 32	MEM 16	MEM 8	MEM 4	MEM 2	MEM 1	CAV 4/4	CAV 2/4	CAV 1/4	CAV 0.5/4
C	ETP 64	ETP 32	ETP 16	ETP 8	ETP 4	ETP 2	ETP 1	FEP 64	FEP 32	FEP 16	FEP 8	FEP 4
D	CMZ 128	CMZ 64	CMZ 32	CMZ 16	CMZ 8	CMZ 4	CMZ 2	PB 16	PB 8	PB 4	PB 2	PB 1
E	CAZ 128	CAZ 64	CAZ 32	CAZ 16	CAZ 8	CAZ 4	CAZ 2	TGC 8	TGC 4	TGC 2	TGC 1	TGC 0.5
F	CTX 128	CTX 64	CTX 32	CTX 16	CTX 8	CTX 4	CIP 8	CIP 4	CIP 2	CIP 1	CIP 0.5	CIP 0.25
G	TZP 256/4	TZP 128/4	TZP 64/4	TZP 32/4	TZP 16/4	TZP 8/4	AK 128	AK 256	AK 64	AK 32	AK 16	AK 8
H	SCF 256/128	SCF 128/64	SCF 64/32	SCF 32/16	SCF 16/8	SCF 8/4	ATM 128	ATM 64	ATM 32	ATM 16	ATM 8	ATM 4

Table note:
All concentration gradients in the above table are expressed in units of μg/mL.

Figure S1.A) Antimicrobial susceptibility results of hvKP4. B) Antimicrobial susceptibility results of 22ZR-42. C) CRE Antimicrobial Susceptibility Testing Panel (2025).

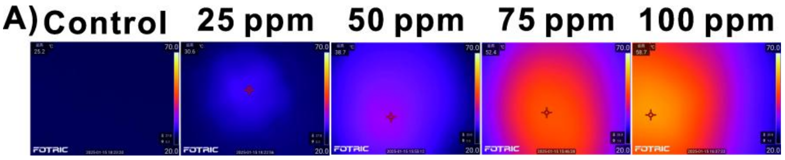

Figure S2. Photothermal infrared thermal images of samples at corresponding concentrations.

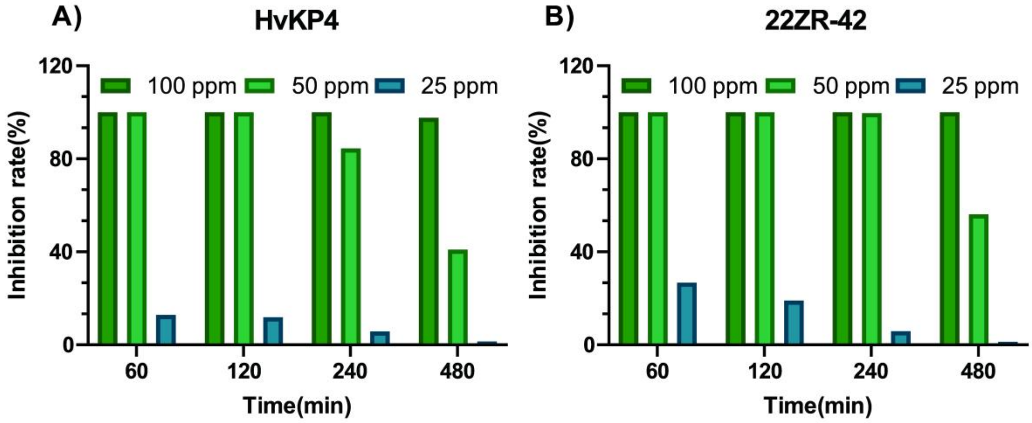

Figure S3.HVKP4 and 22ZR-42 inhibition rates after treatment with different concentrations of Fe/PPy + NIR，***P < 0.001，**P < 0.01,*P < 0.05

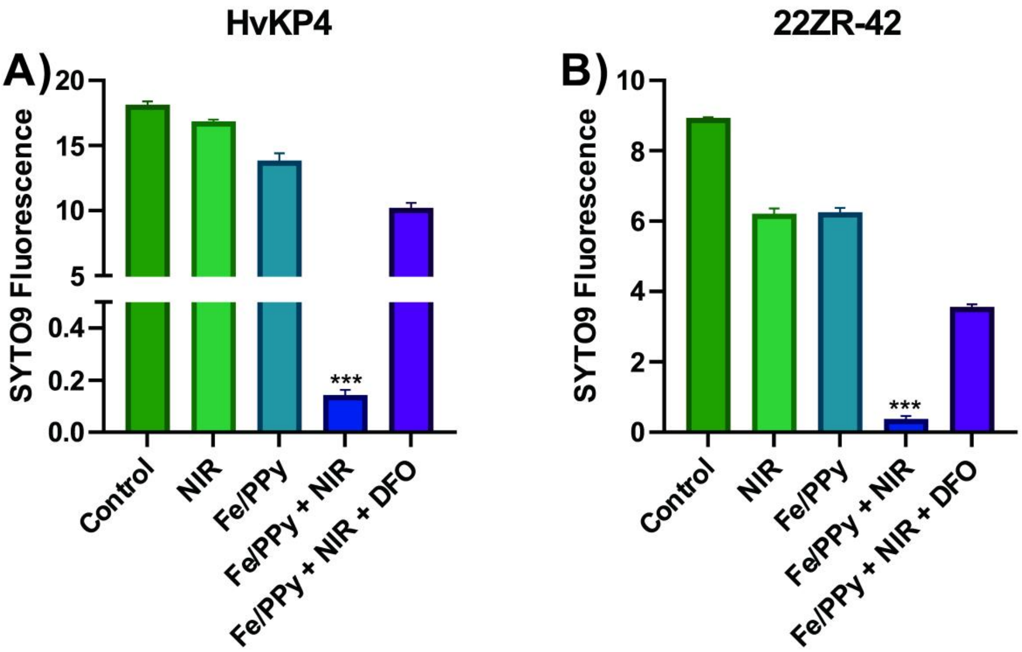

Figure S4.HVKP4 and 22ZR-42 Fluorescence intensity of SYTO9 under different conditions(Control, NIR, Fe/PPy, Fe/PPy + NIR, Fe/PPy + NIR + DFO)， ***P < 0.001，**P < 0.01,*P < 0.05

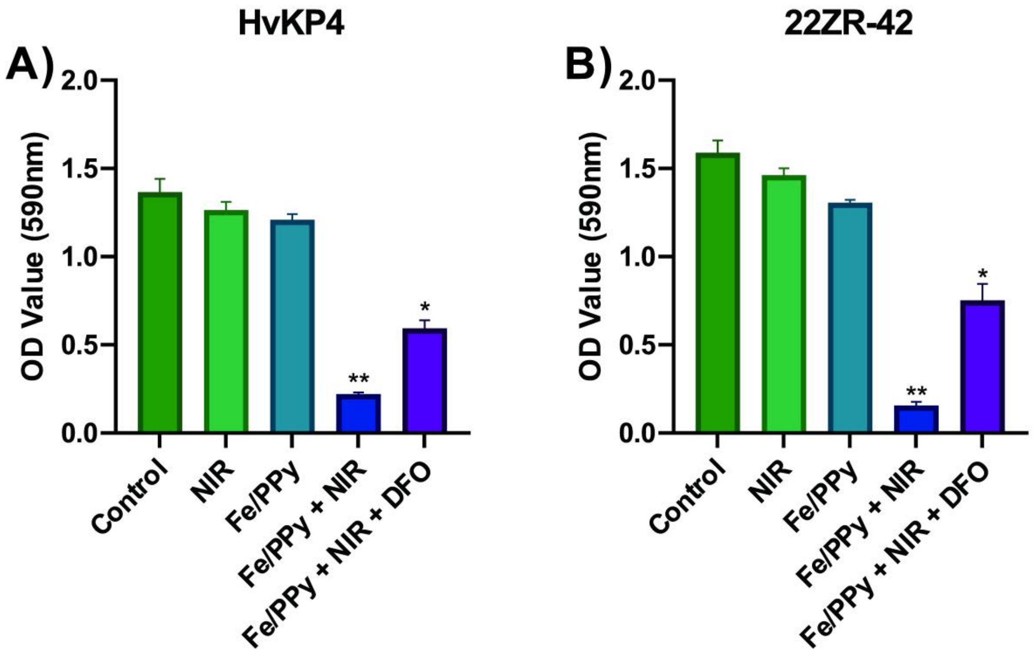

Figure S5. Crystal violet staining OD_{590} values of biofilm formation in HvKP4 and 22ZR-42 under different conditions(Control, NIR, Fe/PPy, Fe/PPy + NIR, Fe/PPy + NIR + DFO).

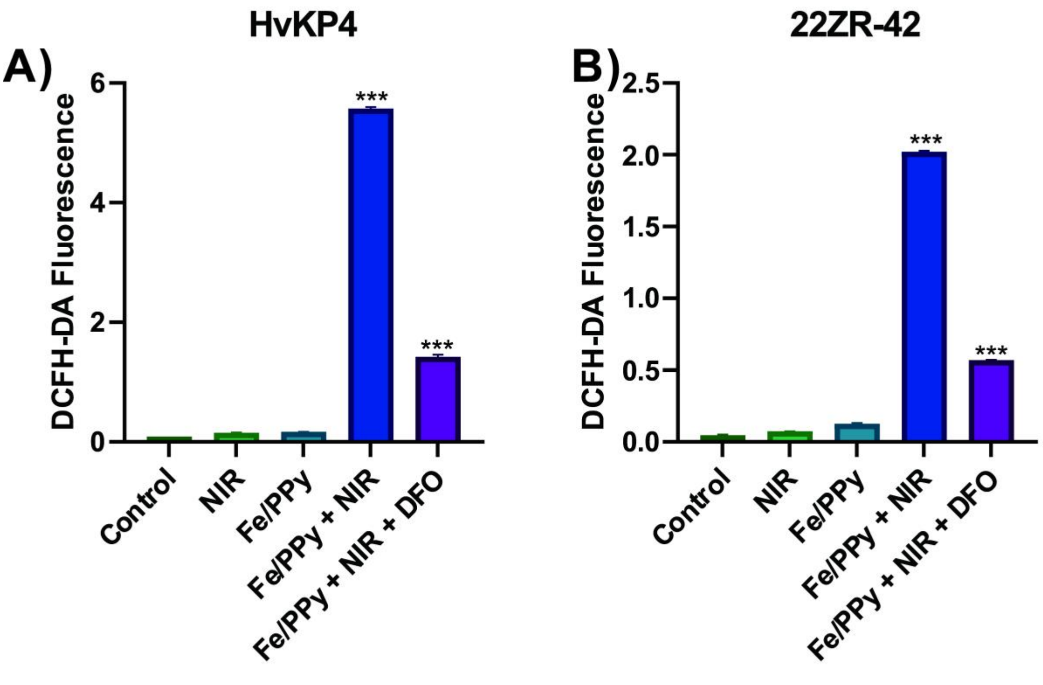

Figure S6.HVKP4 and 22ZR-42 Fluorescence intensity of DCFH-DA under different conditions(Control, NIR, Fe/PPy, Fe/PPy + NIR, Fe/PPy + NIR + DFO).

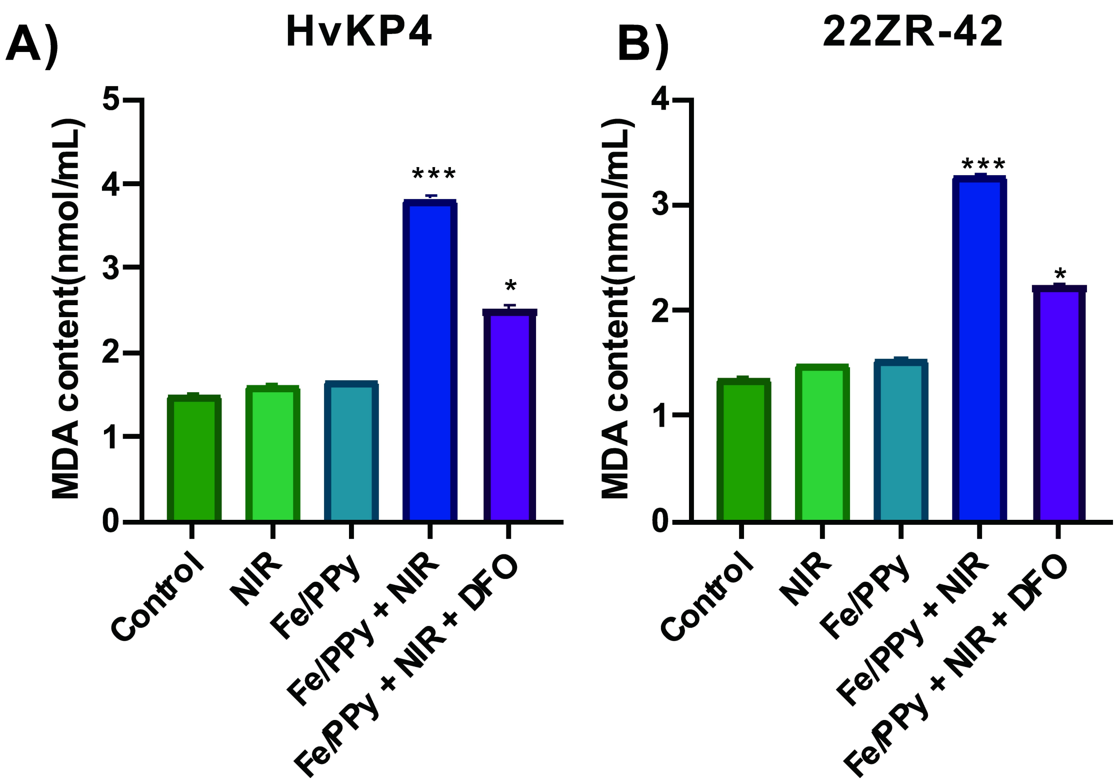

Figure S7.MDA content in HVKP4 and 22ZR-42 after different conditions(Control, NIR, Fe/PPy, Fe/PPy + NIR, Fe/PPy + NIR + DFO).

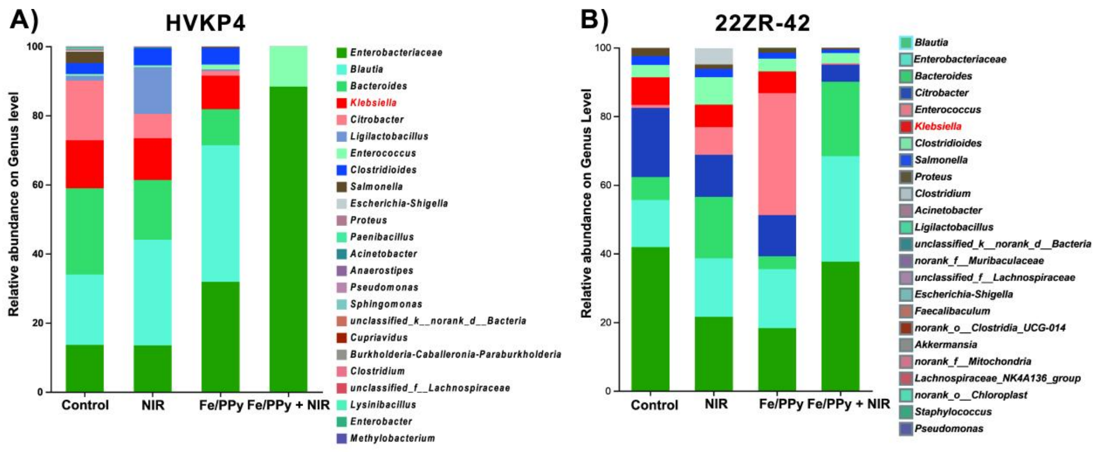

Figure S8. Comparative analysis of microbial community composition between hvKP4 and 22ZR-42.

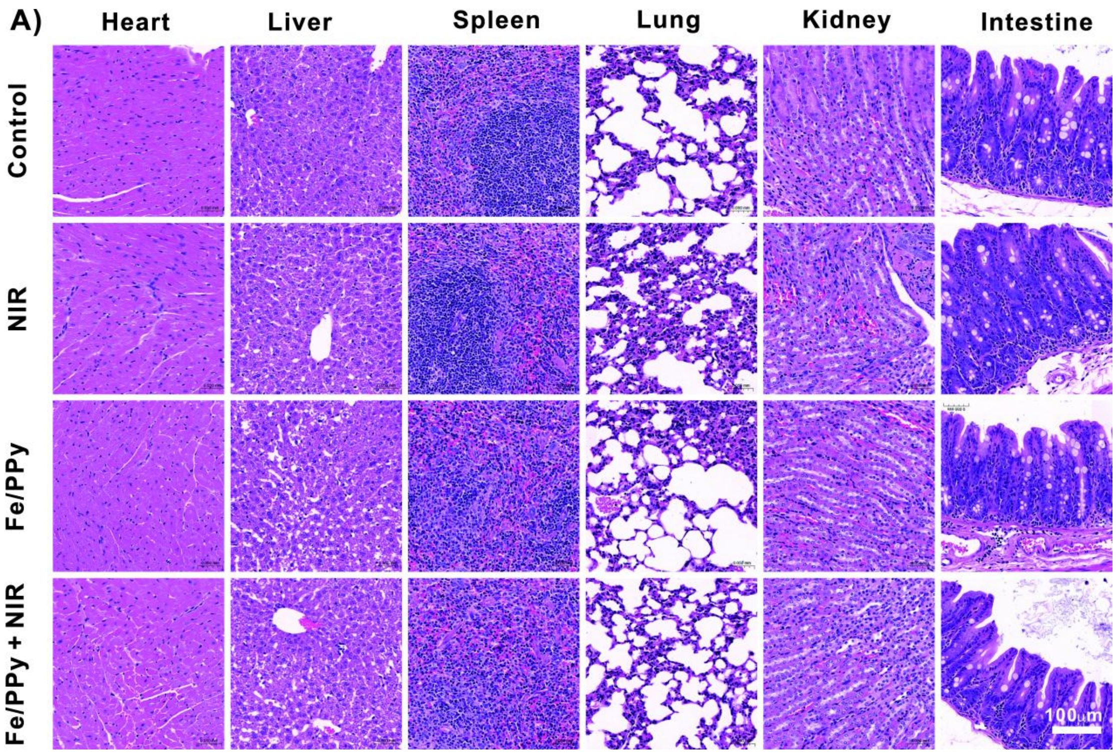

Figure S9.H&E staining results of mouse tissues from different treatment groups(Control, NIR, Fe/PPy, Fe/PPy + NIR, Fe/PPy + NIR + DFO).
